# Transcriptome Sequencing and Gene Expression Analysis of *Trichoderma brevicompactum* under Different Culture Conditions

**DOI:** 10.1371/journal.pone.0094203

**Published:** 2014-04-07

**Authors:** Xu-Ping Shentu, Wei-Ping Liu, Xiao-Huan Zhan, Yi-Peng Xu, Jian-Feng Xu, Xiao-Ping Yu, Chuan-Xi Zhang

**Affiliations:** 1 Zhejiang Provincial Key Laboratory of Biometrology and Inspection & Quarantine, College of Life Sciences, China Jiliang University, Hangzhou, China; 2 Institute of Insect Science, Zhejiang University, Hangzhou, China; 3 Arkansas Biosciences Institute and College of Agriculture, Arkansas State University, Jonesboro, Arkansas, Untied States of America; Institute for Plant Protection (IPP), CNR, Italy

## Abstract

**Background:**

*Trichoderma brevicompactum* is the *Trichoderma* species producing simple trichothecenes-trichodermin, a potential antifungal antibiotic and a protein synthesis inhibitor. However, the biosynthetic pathway of trichodermin in *Trichoderma* is not completely clarified. Therefore, transcriptome and gene expression profiling data for this species are needed as an important resource to better understand the mechanism of the trichodermin biosynthesis and provide a blueprint for further study of *T. brevicompactum*.

**Results:**

In this study, *de novo* assembly of the *T. brevicompactum* transcriptome using the short-read sequencing technology (Illumina) was performed. In addition, two digital gene expression (DGE) libraries of *T. brevicompactum* under the trichodermin-producing and trichodermin-nonproducing culture conditions, respectively, were constructed to identify the differences in gene expression. A total of 23,351 unique transcripts with a mean length of 856 bp were obtained by a new Trinity *de novo* assembler. The variations of the gene expression under different culture conditions were also identified. The expression profiling data revealed that 3,282 unique transcripts had a significantly differential expression under the trichodermin-producing condition, as compared to the trichodermin-nonproducing condition. This study provides a large amount of transcript sequence data that will contribute to the study of the trichodermin biosynthesis in *T. brevicompactum*. Furthermore, quantitative real-time PCR (qRT-PCR) was found to be useful to confirm the differential expression of the unique transcripts.

**Conclusion:**

Our study provides considerable gene expression information of *T. brevicompactum* at the transcriptional level,which will help accelerate the research on the trichodermin biosynthesis. Additionally, we have demonstrated the feasibility of using the Illumina sequencing based DGE system for gene expression profiling, and have shed new light on functional studies of the genes involved in *T. brevicompactum* biosynthesis.

## Introduction

The filamentous fungus *Trichoderma brevicompactum* was firstly identified and confirmed in 2004 [1]. According to the current study, *T. brevicompactum* is the *Trichoderma* species producing simple trichothecenes-trichodermin [2]–[4], a potent antifungal antibiotic and a protein synthesis inhibitor in mammalian cells [5]–[6]. *T. brevicompactum* (IBT 9471) had been mistaken for *T. harzianum* (ATCC 90237) on the basis of considerable shared micromorphology, and later this IBT 9471 strain was reclassified as *T. arundinaceum* based on the phylogenetic lineage within the morphological species *T. brevicompactum* and trichothecene production feature [1], [3], [4]. Now by using a polyphasic (morphological characters, sequence analysis and substrate assimilation patterns) approach, *T. brevicompactum* was recognized as a member of *Brevicompactum* clade, a separate lineage in *Trichoderma*/*Hypocrea*. This includes *T. brevicompactum* and 4 other species −*T. arundinaceum*, *T. turrialbense*, *T. protrudens* and *Hypocrea rodmanii*. So, the reported studies on *T. brevicompactum* are very limited, as compared to those of the other *Trichoderma* species such as *T. viride* and *T. harzianum* [4].

Trichothecenes are a large group of sesquiterpenoid-derived secondary metabolites, and have mainly been isolated from species of *Fusarium* and certain other fungal genera like *Stachybotrys*, *Myrothecium*, *Trichoderma* and *Trichothecium* [2], [7]. The trichothecene biosynthetic pathway has been studied in detail in *Fusarium* species [8]–[11]. However, this biosynthetic pathway is not completely clarified in *Trichoderma* genus. Yet, there are inconsistent results reported regarding the trichothecene biosynthetic pathway in *Trichoderma* [4], [12]. Although *Trichoderma* is a fungal genus of high biotechnological value, the genomic resources available for *Trichoderma*, especially *T. brevicompactum*, are scarce [13]–[14]. It is necessary to obtain more genetic data for further study of *T. brevicompactum* and elucidating the biosynthesis mechanism of trichodermin in this species.

An isolate 0248 was earlier screened from the plant endophytic fungi, and morphologically and molecularly identified as *T. brevicompactum*. Our bioassay showed that *T. brevicompactum* 0248 contains a high antifungal activity against phytopathogens such as *Rhizoctonia solani*, *F. oxysporum*. In further study, it was found that *T. brevicompactum* 0248 was able to synthesize the secondary metabolite trichodermin; the production of the trichodermin was high in a dextrose beef extract medium, but no trichodermin was produced in a α-lactose peptone medium. There was no significant difference in the harvested biomass of *T. brevicompactum* 0248 grown in the above two media; the dry weight of the mycelia was 5.82 g/L in the trichodermin-producing medium and 5.76 g/L in the trichodermin-nonproducing medium after 72 h of fermentation.

Recent research has shown that the high-throughput RNA-sequencing (RNA-Seq) technology is a powerful and cost-efficient tool for transcriptome analysis [15]–[17]. For example, Illumina sequencing technology offers millions of sequence reads from a single instrument run, and generates 150–200 Gigabases of 100 base pair (bp) paired-end sequence in roughly 9.5 days, where the “paired-end” refers to the sequences obtained from the respective opposite ends of a single DNA molecule [18]. It has been shown that this volume of sequencing data can provide ample read coverage for *de novo* transcriptome assembly as well as for gene expression analysis and gene discovery [19]. However, high-throughput DNA sequencing techniques have not yet been applied to the *T. brevicompactum* research.

In this study, over two billion bases of high-quality DNA sequence were generated using the Illumina technology and subsequently assembled. A total of 23,351 unique transcripts were finally obtained from the *T. brevicompactum* 0248 transcriptome. Furthermore, the gene expression profiles of *T. brevicompactum* 0248 grown in the trichodermin-producing and trichodermin-nonproducing conditions were compared using a digital gene expression system. The assembled, annotated transcriptome and the gene expression profiles provided an invaluable resource for the identification of the genes involved in the trichodermin biosynthesis in *T. brevicompactum*.

## Materials and Methods

### Microorganism

*T. brevicompactum* 0248 was isolated from the healthy stems of garlic (*Allium sativum*). The isolate was maintained on a PDA slant medium containing potato- dextrose agar at 4°C until used.

### Culture conditions

Spores of *T. brevicompactum* 0248 (1 mL, 1.0×10^6^ conidia/mL) were inoculated into 300 ml Erlenmeyer flasks containing 60 ml each of the two different media as follows: 1) an α-lactose peptone trichodermin-nonproducing medium containing 1% (w/v) α-lactose, 1% (w/v) peptone, 0.1% (w/v) monopotassium phosphate and 0.05% (w/v) magnesium sulfate; and 2) a dextrose beef extract trichodermin-producing medium containing 0.8% (w/v) dextrose, 1% (w/v) beef extract and 0.2% (w/v) sodium chloride. The flasks were shaken on a rotary shaker at 150 rpm and 28±1°C for 4 days. The mycelia were harvested by filtration for immediate total RNA extraction.

### Trichodermin detection

The trichodermin was quantified by gas chromatography (GC) on an Agilent 6890N equipped with an HP-5 capillary column (5% phenyl methyl siloxane, 30 m×320 μm×0.25 μm) and an FID detector. The injector and the detector temperature were set at 250°C and 270°C, respectively. The column temperature was raised by program with an initial temperature of 100°C maintaining for 5 min, and an increase at a rate of 10°C/min up to 260°C maintaining for 5 min. The flow rate of the nitrogen carrier gas was 1.5 mL/min [20].

### cDNA library preparation and Illumina sequencing for transcriptome analysis

The Axygen Total RNA Isolation kit (Union city, CA) was used to extract the total RNA from *T. brevicompactum* 0248 grown in the trichodermin-producing medium or the trichodermin-nonproducing medium. The two total RNA samples were pooled (w/w, 1:1) for transcriptome analysis to obtain the complete gene expression information. According to the Illumina manufacturer's instructions, poly (A)^+^ mRNA was isolated from the pooled total RNA sample using Oligo (dT) magenetic beads. Fragmentation buffer was added for interrupting mRNA to short fragments. Taking these short fragments as templates, a random hexamer-primer was used to synthesize the first-strand cDNA. The second-strand cDNA was synthesized using buffer, dNTPs, RNase H and DNA polymerase I. After the end reparation and ligation of adaptors, the products were amplified by PCR and purified using the QIAquick PCR Purification Kit (Qiagen, Valencia, CA) to create the final cDNA library.

The cDNA library was sequenced by Beijing Genomics Institute (BGI)-Shenzhen (Shenzhen, China) on an Illumina HiSeq2000. Using Solexa GA pipeline 1.6, the raw reads from the images were generated. By removing adaptor sequences, empty reads and low quality reads, the raw reads were cleaned. The cleaned reads with an identity value of 95% and a coverage length of 200 bp were assembled using the Trinity *de novo* software and clustered using the TGICL and Phrap Clustering tools to get sequences that could not be extended at either end. The obtained sequences were defined as unique transcripts (or unigenes) [21]–[23]. All the raw transcriptome data have been deposited in the NCBI Short Read Archive (SRA).

The generated unigenes were firstly analyzed by searching the NCBI nr database with the BLASTX algorithm (http://www.ncbi.nlm.nih.gov/). Functional annotation by Gene Orthology (GO) was analyzed using the Blast2go (http://www.blast2go.org/) software. The COG and KEGG Orthology (KO) annotations of the unigenes were performed using the Blastall software against the Cluster of Orthologus Groups (COG) database and the Kyoto Encyclopedia of Genes and Genomes (KEGG) database. HMMER(http://hmmer.org/)was used to obtain the domain-based annotation by the Pfam (http://Pfam.sanger.ac.uk) terms [24].

### Digital gene expression (DGE) library preparation and sequencing

Total RNA was extracted from *T. brevicompactum* 0248 under the trichodermin- producing condition or the trichodermin-nonproducing condition using the Axygen Total RNA Isolation kit (Union City, CA). Poly(A)^+^ RNA was purified from 6 μg of total RNA using Oligo(dT) magnetic beads. The double-stranded cDNA was synthesized using the Oligo(dT) as a primer and then digested with the restriction enzyme *Nla*III, which recognizes and cleave the CATG sites. The Illumina adaptor 1 (sense: 5′ACACTCTTTCCCTACACGA CGCTCTTCCGATC3′) was ligated to the sticky 5′ end of the digested bead-bound cDNA fragments. The bead-bound cDNA fragments with the adaptor 1 were then digested by *Mme*I at the 3′ end to release the tag fragments from the Oligo(dT) beads. The Illumina adaptor 2 (sense: 5′ GATCGGAA GAGCGGTTCAGCAGGAATGCCGAG3′) was then ligated to the 3′ ends of the tags, generating the tags with different adaptors at two ends to form a tag library. The library was amplified by linear PCR for 15 cycles, and the 95 bp fragments were purified from 6% PAGE gels. After denaturation, the single-stranded molecules were attached to the Illumina sequencing chip for sequencing with the adaptor 1 used as the primer. Each tunnel of chip generated millions of raw tags with a length of 35 bp. All the raw tag data have been deposited in NCBI SRA.

### Analysis and mapping of DGE tags

To map the DGE tags, the sequenced raw data were transformed into clean tags by removing the low quality tags (tags with unknown nucleotide “N”), empty tags (no tag sequence between the adaptors) and tags with only one copy number (which might result from sequencing errors). For tag annotation, the clean tags containing CATG and 21 bp tag sequences were mapped to our transcriptome reference database, and only 1 bp mismatch was considered. The clean tags were designated as unambiguous clean tags. For gene expression analysis, the number of unambiguous clean tags for each gene was calculated and then normalized to TPM (number of transcripts per million clean tags).

### Evaluation of DGE libraries

According to the method described by Audic and Claverie [25], a statistical analysis of the frequency of each tag in the different DGE libraries was performed to compare the differences in gene expression. The false discovery rate (FDR) was used to determine the threshold P-value in multiple testing and analysis [25]. A FDR< 0.001 and an absolute value of the log2 ratio>1 were used as the threshold to determine the statistical significant difference in gene expression [26]. For the differentially expressed genes, GO and KO enrichment analyses were performed. Enriched P values were calculated on the base of the hypergeometric test as follows:
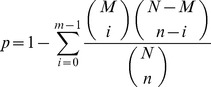



Where N is the number of all genes with GO/KO annotation; n is the number of differentially expressed genes in N; M is the number of all genes in each GO/KO term; m is the number of differentially expressed genes in each GO/KO term. For GO enrichment analysis, all of the P-values were determined with Bonferroni correction. The corrected P-value (<0.05) was selected as a threshold to determine the significant enrichment of the gene sets. On the contrary, for KO enrichment analysis, a FDR<0.05 was used as the threshold to judge the significant enrichment of the gene sets [25].

### Quantitative real-time PCR validation

Three mycelia samples of *T. brevicompactum* under the trichodermin-producing or trichodermin-nonproducing conditions were obtained and their total RNAs were extracted as described for the DGE library preparation. Then reverse transcription of each RNA sample was performed to get cDNA using the PrimeScript RT reagent Kit (TaKaRa) with an Oligo dT primer. The cDNA was used for Quantitative real-time PCR (qRT-PCR) analysis. The qRT-PCR was performed in triplicates using the SYBR Premix Ex Taq Kit (TaKaRa). The specificity of the SYBR green PCR signal was further confirmed by a melting curve analysis. The *β-tubulin* gene of *T. brevicompactum* 0248 was used as the reference gene. Primers used for this study were shown in Table S1. The normalized fold changes of the target gene mRNA expression were expressed as 2^−ΔΔCT^. In order to reduce the error, the PCR reaction conditions for both the reference gene and target genes were optimized by changing annealing temperature and annealing time, and their amplication efficiencies were within the range of 95%∼100%.

## Results

### Illumina sequencing and sequence assembly

**Figure 1:**
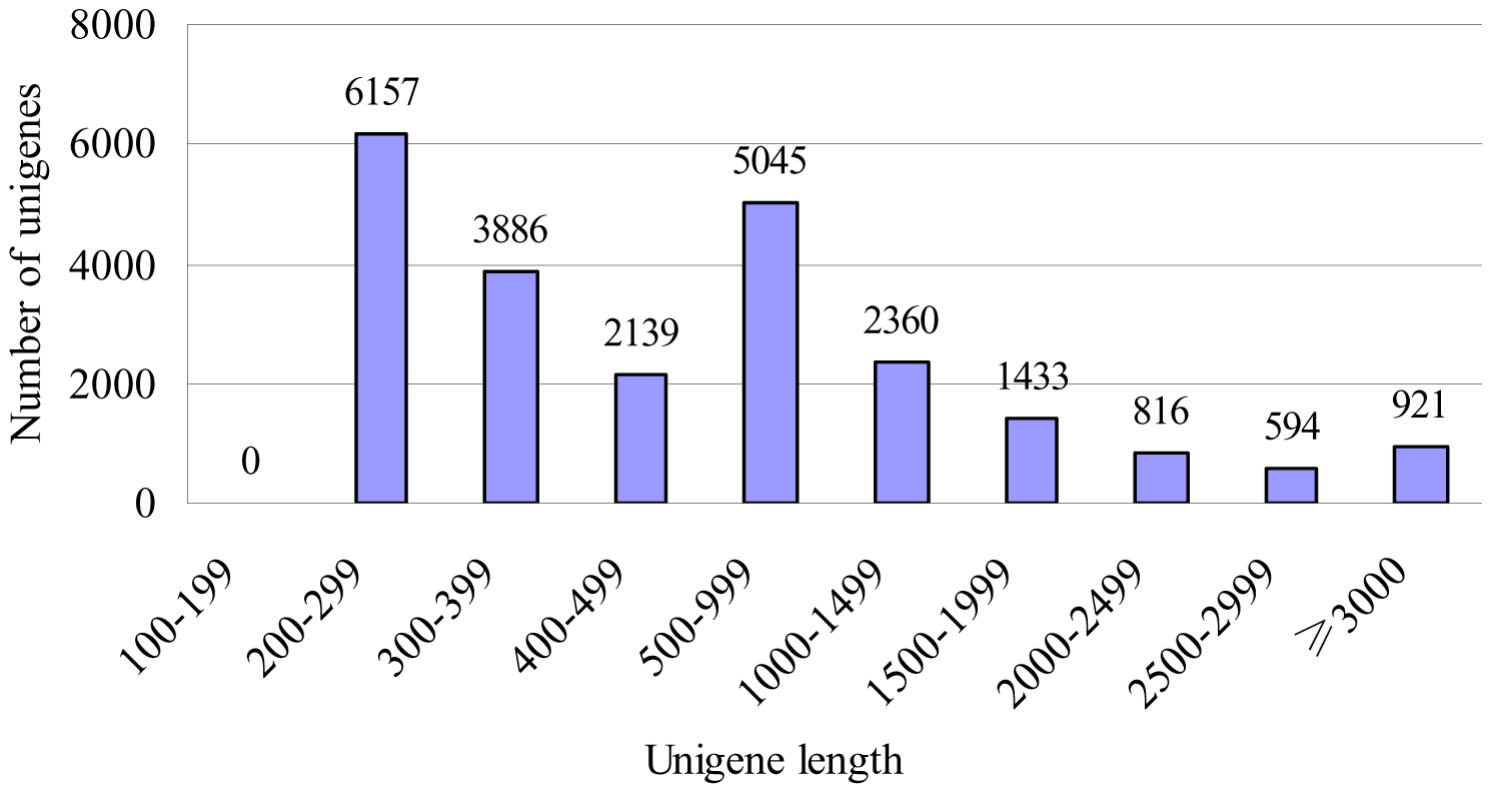
Unigenes size distribution.

To obtain an overview of *T. brevicompactum* 0248 gene expression profiles, a cDNA sample was prepared from the fungus grown under both the trichodermin-producing and trichodermin-nonproducing conditions and sequenced using the Illumina sequencing platform. A total of 25,816,102 reads (accumulated length of 2,323,449,180 bp; SRA accession number SRX209600) were generated through the Illumina sequencing. All reads were assembled *de novo* by the Trinity, which generated 23,922 contigs with a mean contig length of 861 bp and a N50 of 1,449 bp. To further join the sequences and remove any redundant sequences, the contigs were clustered using the TGICL and Phrap tools [21]. A total of 23,351 unigenes were produced, with a mean length of 856 bp and a N50 of 1,446 bp. The size distribution of these unigenes was shown in Figure 1. Most of the unigenes (6,124) were longer than 1000 bp (Figure 1). To prove the quality of the sequencing data, we randomly selected 15 unigenes and designed 15 pairs of primers for RT-PCR. It showed that 14 out of 15 primer pairs resulted in a band of the expected size on an agarose gel, and the identity of all the fourteen PCR products was confirmed by the Sanger sequencing (data not shown).

### Annotation of predicted proteins

**Figure 2:**
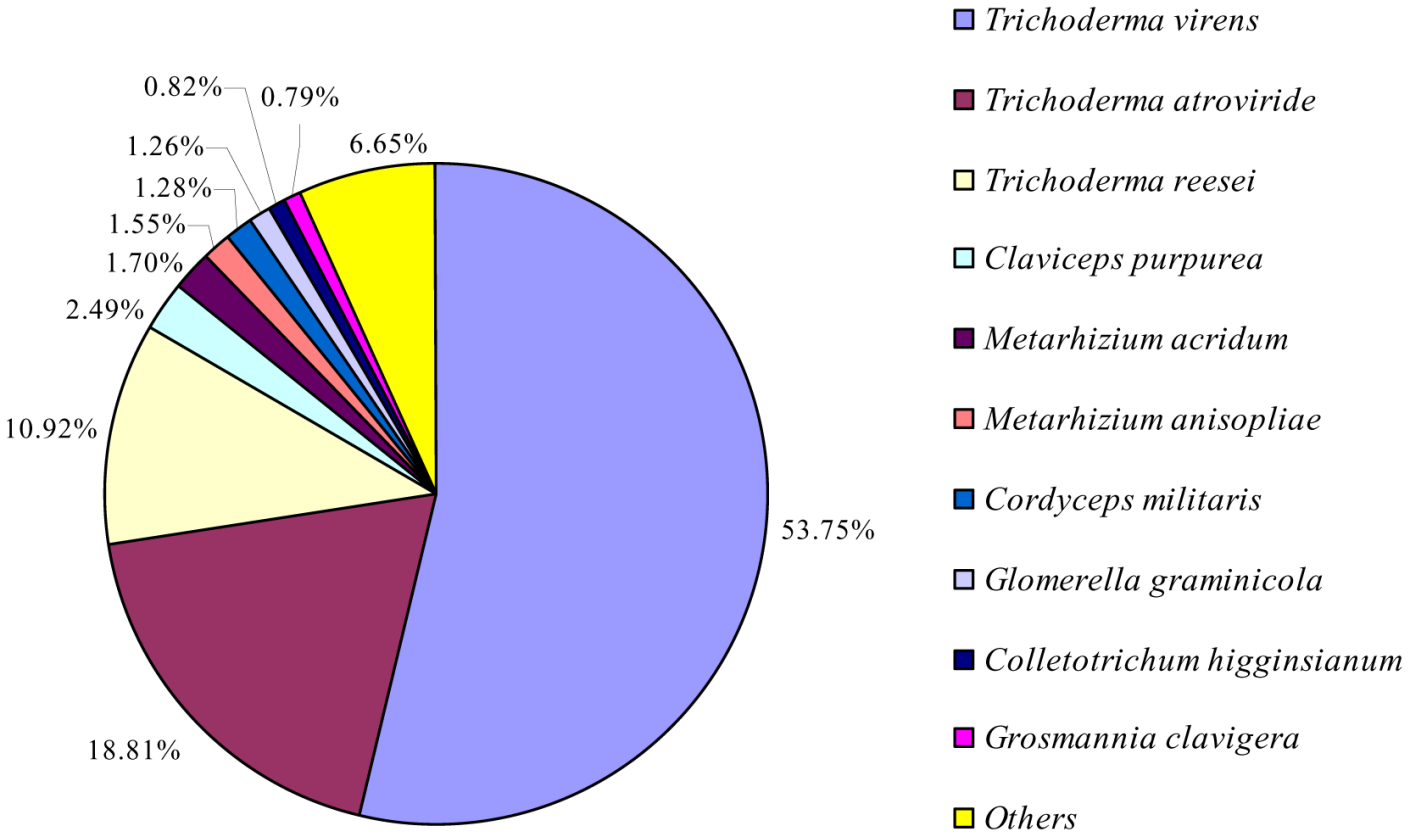
Species distribution of the BLASTX results. Unigenes were searched using BLASTX against the nr protein database with a cutoff E value<10^−5^. Different colors represent different species. We used the first hit of each unigene for analysis.

For annotation, unigenes were first searched using BLASTX against the non-redundant (nr) NCBI protein database with a cut-off E-value of 10^-5^. A total of 19,317 (82.72% of all distinct sequences) unigenes were annotated and the results were shown in Table S2. The species distribution of the best match result for each sequence was shown in Figure 2. The *T. brevicompactum* 0248 sequences showed a 53.75% match with the *T. virens* sequences, followed by the sequences of *T. atroviride* (18.81%), *T. reesei* (10.92%), *Claviceps purpurea* (2.49%) and *Metarhizium acridum* (1.70%). The distribution of unigene numbers of *T. brevicompactum* with the orthologues in *T. virens*, *T. atroviride* and *T. reese* was shown in Figure S1. Among the 19,317 annotated unigenes, *T. brevicompactum* shared 17,506, 15,850 and 14,736 homologous ones with *T. virens*, *T. atroviride* and *T. reesei*, respectively. The vast majority of the unigenes of *T. brevicompactum* (13,681) could be mapped to the predicted proteins of all the other three *Trichoderma* species. However, there were still 1,027 unigenes having no true orthologues in any of the other three species, which could make *T. brevicompactum* unique and possibly enable it to synthesize the trichodermin. It was found that among the 1,027 unigenes, many of them are homologous to those encoding acetyltransferase and cytochrome P450 monooxygenase, indicating these two enzymes are important to trichothecene biosynthesis in *Trichoderma*. Furthermore, the 3,834 unigenes that could not be annotated using BLASTX against the non-redundant (nr) NCBI protein database were not homologous with the other three species. Although the number of unigenes produced from the *T. brevicompactum* transcriptome assembly in our study was bigger than the gene numbers predicted from the genome sequencing of *T. virens*, *T. atroviride* and *T. reesei*,this Venn diagram (Figure S1) showed the difference in gene sets between *T. brevicompactum* and the other three species in some ways.

### GO, KEGG, COG and Pfam classification

**Figure 3:**
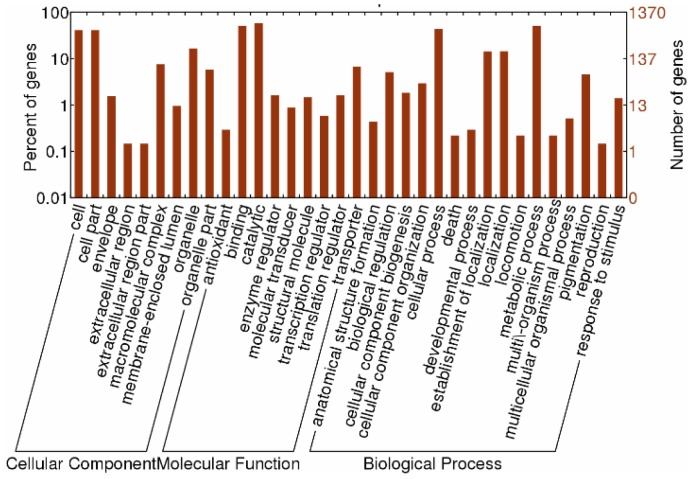
GO categories of the unigenes. The unigenes were mainly annotated in three categories: cellular components, molecular functions and biological processes. The right y-axis denotes the number of genes in a category. The left y-axis denotes the percentage of a specific category of genes in the main category.

**Figure 4:**
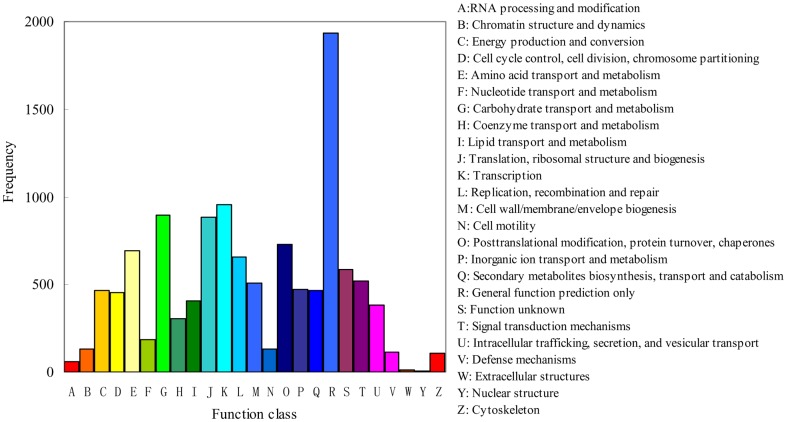
Histogram of the clusters of orthologous groups (COG) classification. Out of 19,317 nr hits, 6,140 unigenes have a COG classification among the 25 categories.

A total of 1,370 unigenes were assigned to one or more GO codes (Table S3) and categorized into 33 functional groups based on sequence homology (Figure 3). In each of the three main categories of the GO classification − biological process, cellular component and molecular function, the terms of ‘metabolic process’, ‘cell’ and ‘cell part’, and ‘catalytic activity’ are dominant, respectively. We also noticed a high percentage of the unigenes from the categories of ‘cellular process’ and ‘binding’, but only a few genes from the terms of ‘multicellular organismal process’, ‘reproduction’, and ‘extracellular matrix’ (Figure 3). At the same time, the unigenes were aligned to the COG database to predict and classify the possible functions. In total, 6,140 unigenes out of 19,317 nr hits had a COG classification (Figure 4). Among the 25 COG categories, the category for ‘general function prediction only’ represents the largest group (1937, 31.55%), followed by the categories of ‘ transcription’ (958, 15.60%) and ‘carbohydrate transport and metabolism’ (897, 14.61%). The categories for ‘nuclear structure’ (6, 0.10%), ‘extracellular structures’ (12, 0.20%) and ‘RNA processing and modification’ (58, 0.94%) represent the smallest groups (Figure 4). To identify the biological pathways that are active in *T. brevicompactum*, 19,317 annotated unigenes were mapped to the reference canonical pathways in Kyoto Encyclopedia of Genes and Genomes (KEGG) [27]. In total, 9,093 unigenes were assigned to 108 KEGG pathways. The pathways mostly represented by the unigenes were metabolic pathways (2326 members), biosynthesis of secondary metabolites (975 members) and RNA transport (448 members). By searching the Pfam-A database using the HMMER (http://hmmer.org/), about 1938 (10.03%) unigenes were annotated to relevant protein families and 1455 families were found. The Pfam-A E-value 1.0 is a threshold. The five most abundant Pfam domains were ‘NAD_binding_8′ (39), ‘DAO’ (39), ‘Pkinase’ (37), ‘Rav1p_C’ (35) and ‘Pyr_redox_2’ (32). More detailed information about the structure and the domain architecture of the annotated proteins are available from the website (http://pfam.janelia.org/). These annotations provide a valuable resource for investigating the specific biological processes, functions and pathways of *T. brevicompactum*.

### DGE library sequencing

An immediate application of our transcriptome sequence data is the gene expression profiling under different culture conditions. Using the DGE method, which generates absolute rather than relative gene expression measurements and avoids many of the inherent limitations of transcriptome analysis, we analyzed the gene expression variations under the different culture conditions of *T. brevicompactum* 0248. We sequenced the two DGE libraries: the trichodermin-producing (SRX207877) and trichodermin-nonproducing (SRX206687) libraries, and generated about 5.4 and 5.5 million raw tags, respectively (Table S4). After the low quality tags were filtered, the total number of the clean tags in both the libraries were over 5 million (Table S4), and in both cases the clean tags accounted for more than 95% of the raw tags (Figure S2). Among the clean tags, the number of the sequences that could be mapped to unigenes were 4.6 million (89.53%) and 3.6 million (65.97%), respectively, in the trichodermin-producing and trichodermin-nonproducing libraries (Table S4).

To evaluate the DGE data, the distribution of the clean tag expression was analyzed (Figure S3). In both the libraries, the highly expressed genes, *i.e.*, those with a copy number more than 100, accounted for greater than 83% of the clean tags, but their distribution of distinct clean tags was less than 7.00%. In contrast, the genes with a low level of expression, *i.e*., those with a copy number less than 5, showed a broad distribution of distinct clean tags (Figure S3).

### Gene expression variations under different culture conditions

To identify the differentially expressed genes of *T. brevicompactum* under the two different culture conditions, the number of the clean tags for each gene was calculated. The genes that were differentially expressed between the two samples were identified using an algorithm developed by Audic and Claverie [25]. The results revealed 3,282 genes with a significantly differential expression (Figure S4). Among them, 1,699 genes were up-regulated and 1,583 were down-regulated under the trichodermin-producing condition, as compared to the culture under the trichodermin-nonproducing condition. Giving insights into 20 of the most differentially up-regulated and 20 of the most down-regulated genes, 11 out of the 20 down-regulated genes have defined functions, and this rate goes up to 18/20 in the most up-regulated genes (Table S5). Only 27.5% of the highly regulated genes are orphan sequences - no homologues found in the NCBI database.

To understand the functions of the differentially expressed genes, all the genes were mapped to terms in the KEGG database and compared with the whole transcriptome background in order to search for the genes involved in the metabolic or signal transduction pathways that were significantly enriched. Among all the genes with the KEGG pathway annotation, 1303 differentially expressed genes were identified in the trichodermin-nonproducing and the trichodermin-producing libraries. In 106 KEGG pathways, specific enrichment of genes was observed in the ‘starch and sucrose metabolism’ pathway, the ‘amino sugar and nucleotide sugar metabolism’ pathway, and the ‘ribosome’ pathway in the trichodermin-producing library compared to the trichodermin-nonproducing one. In addition, GO enrichment analysis of the functional significance was also applied in the gene expression profiling analysis. In each of the three main categories of the GO classification (biological process, cellular component and molecular function), unigenes with a significantly differential expression were mainly enriched with the terms of ‘Metabolic process’ and ‘cellular process’, ‘Cell’ and ‘Cell part’, and ‘binding’ and ‘Catalytic activity’, respectively. Interestingly, we also found the unigenes homologous to those involved in the trichothecene biosynthesis in *Fusarium* and *T. arundinaceum* (e.g., *tri4* and *tri5*) were up-regulated under the trichodermin-producing condition. Certainly, these unigenes are also highly homologous to the *T. brevicompactum tri* genes published in the literature [12]. In addition, many other unigenes participating in the terpenoid backbone biosynthesis were also found, for example, the genes encoding a farnesyl-pyrophosphate synthetase, a HMG-CoA reductase, an isopentenyl- pyrophosphate isomerase, and a hydroxy-methylglutaryl-CoA reductase [28].

### Gene expression analysis and qRT-PCR validation

**Figure 5:**
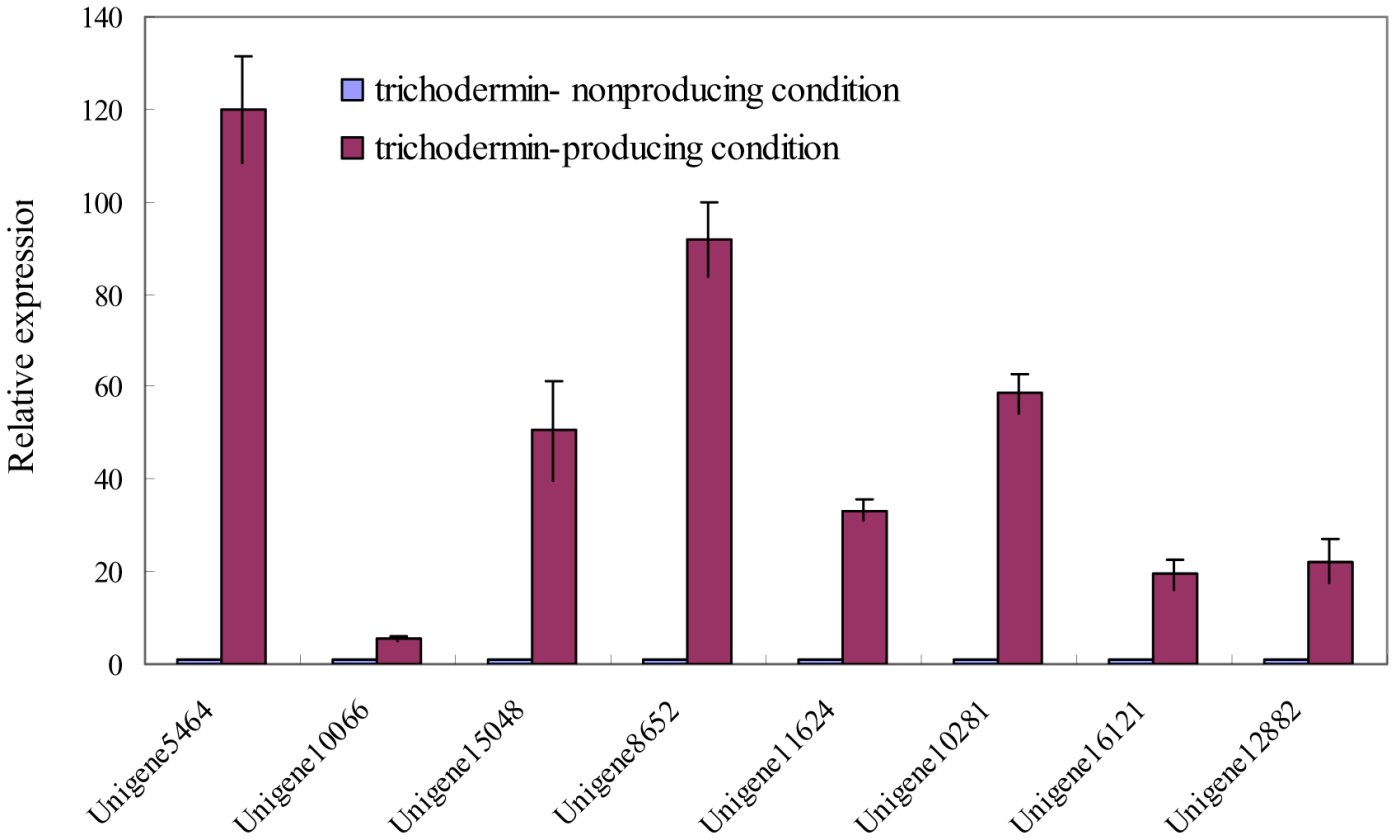
Quantitative PCR (qPCR) analysis of several unigenes in *T. brevicompactum* 0248 under the trichodermin-nonproducing or trichodermin-producing condition. Transcription levels of the unigenes were normalized to the *β-tubulin* gene. Data represent the mean and standard errors (n = 3).

Eight unigenes with potential roles in the trichodermin synthesis or differential expression under different culture conditions of *T. brevicompactum* 0248 were chosen for validation using qRT-PCR(Figure 5). Unigenes 5464 and 10066, which are homologous to the *tri5* and *tri4* genes involved in the trichothecene biosynthesis in *Fusarium* and *T. arundinaceum*, were highly expressed under the trichodermin-producing condition, as compared to under the trichodermin-nonproducing condition. The expression levels of unigenes 8652 and 15048, which can not be annotated through BLASTX against the non-redundant (nr) NCBI protein database, were also significantly up-regulated under the trichodermin-producing condition, as compared to under the trichodermin-nonproducing condition. In addition, the expression levels of four other unigenes (unigene11624, 10281, 16121 and 12882), which had a significant differential expression under the trichodermin-producing condition as compared to the trichodermin-nonproducing condition, were also validated. In general, the trends of down- or up- regulated transcription of the different unigenes by the qRT-PCR analysis were consistent with those of the DGE expression profiling analysis, indicating the reliability of the DGE expression profiling analysis.

## Discussion

*Trichoderma* species are widely used as biocontrol agents for plant pathogenic fungi. The generally accepted mechanisms of *Trichoderma* biocontrol include mycoparasitism, competition, and antibiosis [29]. Both *T. brevicompactum* and *T. arundinaceum* can produce trichothecenes such as trichodermin and harzianum A (HA) with the antibiotic and antitumor activities, respectively [12]. Since the biosynthetic pathway of trichothecenes in *Trichoderma* is not clear, the transcriptome sequencing and gene expression analysis of *T. brevicompactum* 0248 under different culture conditions were carried out in this study to improve our understanding about the secondary metabolism of *T*. *brevicompactum*. This will help further increase the trichodermin production through metabolic engineering.

Based on the transcriptome data, we found that *T*. *brevicompactum* has a high similarity to other *Trichoderma* species such as *T. virens*, *T. atroviride* and *T. reesei* according to the BLAST annotation. This is easy to understand because they belong to the same genus in the taxonomic classification. The Venn diagram (Figure S1) showed the distribution of the orthologues of these four species and also revealed the difference between *T. brevicompactum* and the other three species. On the other hand, *T. reesei* does not have known biocontrol abilities, but it is widely used in the industry [13]. In contrast, *T. virens* and *T. atroviride* are widely used biocontrol species, but they do not synthesize trichodermin [14]. Certainly, genome sequencing and analysis of these three *Trichoderma* species will provide valuable genetic information for *Trichoderma* study. Because *T. brevicompactum* shows different biological characteristics and functions from those of the other three species, it deserves further study.

Comparing the COG classification results of *T. brevicompactum* with those of the other three *Trichoderma* species (*T. reesei*, *T. virens* and *T. atroviride*) [30], there was a significant difference in gene numbers in various functional categories (Table S6). Among the 25 COG categories, the cluster for ‘general function prediction only’ represents the largest group in *T*. *brevicompactum*, followed by the cluster for ‘transcription’ and ‘carbohydrate transport and metabolism’. Similarly, the cluster for ‘general function prediction only’ represents the largest group in other three *Trichoderma* species, but the second and third largest groups are ‘posttranslational modification, protein turnover, chaperones’ and ‘signal transduction mechanisms’, respectively. In addition, the cluster for ‘nuclear structure’ represents the smallest group in *T*. *brevicompactum*. In contrast, the “cell motility” is the smallest group in other three *Trichoderma* species. The difference in COG classification between *T*. *brevicompactum* and the other three *Trichoderma* species correlates with their different biocontrol abilities or industrial value. It was reported that *T. brevicompactum* is the *Trichoderma* species producing simple trichothecenes–trichodermin [2]. According to the analysis of KEGG pathways [30], no gene was found to participate in the sesquiterpenoid biosynthesis metabolic pathway in *T. reesei*, *T. virens* and *T. atroviride* so far. On the contrary, many unigenes that are homologous to *tri4* and *tri5* (genes involved in the sesquiterpenoid biosynthesis) were identified. This may explain why *T. reesei*, *T. virens* and *T. atroviride* can not synthesize the trichodermin, a sesquiterpenoid compound.

To better understand the gene expression of *T*. *brevicompactum*, we constructed the cDNA libraries under the different culture conditions for transcriptome analysis. We also created two DEG libraries to analyze the gene expression patterns under the different culture conditions. So far, the studies on many taxonomy genes, such as the 18S rRNA gene and the translation elongation factor 1 alpha gene, have been reported in *T*. *brevicompactum*. However, the study on genes related to trichothecenes biosynthesis in *T*. *brevicompactum* is very limited. Our transcriptome and gene expression profiling data will greatly enrich the current *T*. *brevicompactum* genetic information and contribute to the further research to identify new genes and the biocontrol mechanisms of *T*. *brevicompactum*.

Although both *Fusarium* and *Trichoderma* can synthesize trichothecenes, sequence and functional analysis revealed that the gene (*tri5*) responsible for the first committed step in trichothecene biosynthesis is located outside the cluster in all the *Trichoderma* species, but inside the cluster in *Fusarium* [12]. Heterologous gene expression analysis revealed that the two *T. arundinaceum* cluster genes (*tri4* and *tri11*) differ in function from their *Fusarium* orthologues. The *Tatri4*- encoded enzyme catalyzes only three out of the four oxygenation reactions that are catalyzed by the orthologous enzyme in *Fusarium*. The *Tatri11*-encoded enzyme catalyzes a completely different reaction (trichothecene C-4 hydroxylation) from that of the *Fusarium* orthologue (trichothecene C-15 hydroxylation) [12]. These results indicate that although some characteristics of the *tri/TRI* cluster have been conserved during the evolution of *Trichoderma* and *Fusarium*, the cluster has undergone marked changes, including gene loss and/or gain, gene rearrangement, and divergence of gene function [12]. To date, more than 200 trichothecenes have been reported [7]–[9]. They are divided into four types (A–D) according to their chemical structures [31]–[32]. Type B trichothecenes such as deoxynivalenol (DON) and nivalenol (NIV) are distinguished from type A by the presence of a keto group at C-8 and are mostly produced by *Fusarium* [9]. Trichodermin and HA synthesized by *T. brevicompactum* and *T. arundinaceum*, respectively, are classified into type A [1], [33]. These two compounds have simpler structures than the *Fusarium* trichothecenes DON, NIV and T-2 toxin. This suggests that fewer genes in *Trichoderma* than in *Fusarium* are involved in the trichothecenes biosynthesis. Certainly, the above analysis also showed there are much difference in the trichothecenes biosynthesis between *Fusarium* and *Trichoderma*. In *Fusarium*, the gene *tri8* encodes trichothecene C-3 deacetylase; *tri101* is responsible for the conversion of isotrichodermol to isotrichodermin (i.e.C-3 acetylation); *tri1* is involved in C-8 hydroxylation; *tri16* is proved to encode an acyltransferase that catalyzes the formation of ester side groups at C-8; *tri11* encodes an isotrichodermin C-15 hydroxylase [9]–[10]. The absence of the C-8, C-3 and C-15 oxygen atoms in the structure of trichodermin and HA suggests that the trichothecene-producing *Trichoderma* strains lack the orthologues of the *Fusarium* trichothecene C-8, C-3 and C-15 oxygenase genes(*tri1*, *tri16*, *tri8*, *tri101* and *tri11)*. It is surprising that no homologous genes, such as *tri1*, *tri16*, *tri8* and *tri101* that are related to the trichodiene synthase in *F. graminearum* and *F. longipes*, were detected in our sequencing data (Table S2). This finding further verifies the quality of our sequencing data, and will certainly facilitate the research on the trichothecene biosynthesis in *T. brevicompactum*.

Two completely different pathways of the *Trichoderma* trichothecenes biosynthesis were speculated according to the reports [4], [12]. In one pathway, the trichodermin was synthesized through three- step reactions with trichodiene as the starting compound. However, the genes responsible for these reactions were not clear in the report (Figure S5) [4]. Cardoza *et al.*(2011) also studied the trichothecene biosynthesis genes in *T. arundinaceum* and deduced the HA biosynthetic pathway (Figure S6) [12]. In this pathway, HA was synthesized from trichodiene through four-step reactions. The *Tatri4*-encoded enzyme catalyzed only three out of the four oxygenation reactions that could be catalyzed by the orthologous enzyme in *Fusarium* [12]. The *Tatri11*-encoded enzyme catalyzed a completely different reaction (trichothecene C-4 hydroxylation) from that of the *Fusarium* orthologue (trichothecene C-15 hydroxylation) [12]. The gene responsible for the last step of reaction− acetylation of the C-4 oxygen is unknown so far. Trichodermin is extremely similar to HA in chemical structure. The only difference is the side group at the C-4 position; trichodermin has an acetyl group while HA has an octa-2,4,6-trienedioic acid. In our results, unigenes that are homologous to the *Tatri4*, *Tatri5* and *Tatri11* genes involved in the sesquiterpenoid biosynthesis were identified. Therefore, it is very likely that the trichodermin and HA share the similar biosynthetic pathways in *T*. *brevicompactum* and *T. arundinaceum*. It is hypothesized that the lateral moietie at the C-4 position of the HA might be added to trichodermol through acetylation with an acyltransferase encoded by the *tri3* gene [12]. However, the orthologue of the *tri3* gene has not been found in the *T. brevicompactum* transcriptome analysis. On the contrary, many unigenes that are homologous to the acyltransferase-encoding genes were found (Table S2). Whether the function of these unigenes is similar or not to that of the *tri3* gene needs to be determined in the further.

We have demonstrated the feasibility of using Illumina sequencing-based DGE system for gene expression profiling, and provided valuable data for future functional studies of the genes involved in *T. brevicompactum* biosynthesis.

## Supporting Information

Figure S1 **Distribution of the unigene numbers of** ***T. brevicompactum*** **with the orthologues in** ***T. virens*** **,** ***T. atroviride*** **and** ***T. reesei*** **.**
(JPG)

Figure S2 **Different components of the raw tags in the two samples.** The percentages of the tags containing N, adaptors, a tag copy number<2, clean tags and raw tags. The numbers in parentheses indicate the percentage of each type of tag accounts for the total raw tags. A: trichodermin-producing condition; B: trichodermin-nonproducing condition.
(TIF)

Figure S3 **Distribution of the total clean tags and the distinct clean tags in the two samples.** The numbers in square brackets indicate the range of copy numbers of each tag category. The data in parentheses indicate the percentage of corresponding tags account for the total clean tags and distinct clean tags. A: trichodermin-producing condition; B: trichodermin-nonproducing condition. (TIF)
(TIF)

Figure S4 **DGE unigenes were up-regulated (red) and down-regulated (green) under the trichodermin-nonproducing or trichodermin-producing condition.**
(TIF)

Figure S5 **The putative pathway of the trichodermin biosynthesis in** ***Trichoderma brevicompactum*** **.**
(TIF)

Figure S6 **The putative pathway of the HA biosynthesis in** ***Trichoderma arundinaceum*** **.**
(TIF)

Table S1 **Primers used in qRT-PCR for validation of the differentially expressed genes.**
(XLS)

Table S2 **Top hits obtained by BLASTX for the unigenes. BLASTX against the NCBI nr database was used with a cut-off E-value of 10^−5^.**
(XLS)

Table S3 **GO annotation of unigenes.**
(XLS)

Table S4 **Tag analysis statistics.**
(DOC)

Table S5 **Top 20 most up-regulated and 20 down-regulated unigenes between the trichodermin-producing condition and the trichodermin-nonproducing condition.**
(XLS)

Table S6 **COG classification comparison of different** ***Trichoderma*** **species.**
(XLS)
