# Supplementary figures and images for: Transcriptome Sequencing and Gene Expression Analysis of Trichoderma brevicompactum under Different Culture Conditions
Source: PLoS One. 2014 Apr 7;9(4):e94203. doi: 10.1371/journal.pone.0094203 (PMC3978026; doi:10.1371/journal.pone.0094203)

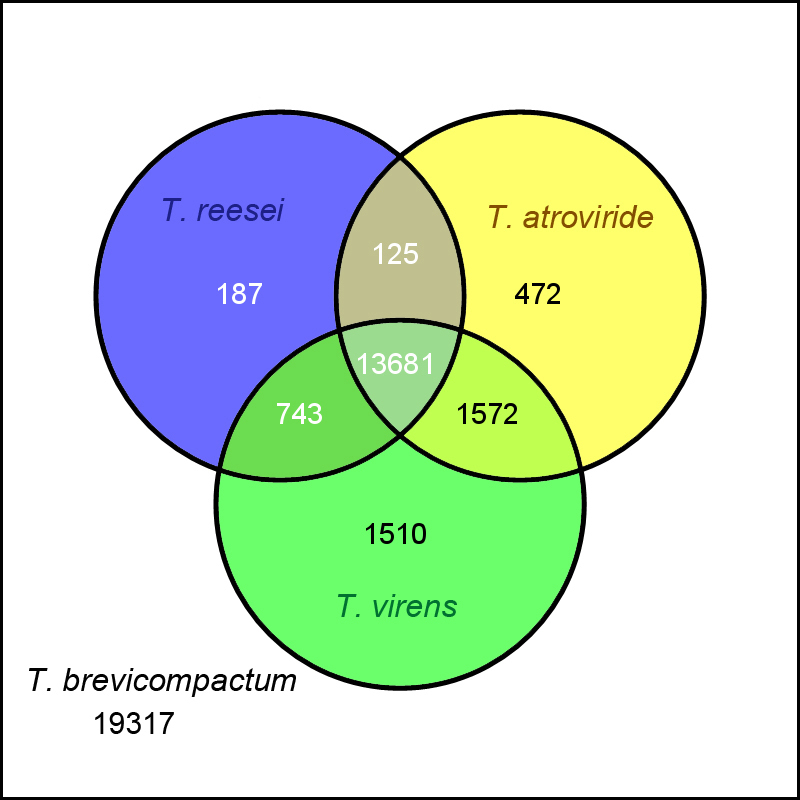

Supplement: Figure S1 — Distribution of the unigene numbers of T. brevicompactum with the orthologues in T. virens , T. atroviride and T. reesei . (JPG) [file pone.0094203.s001.jpg]

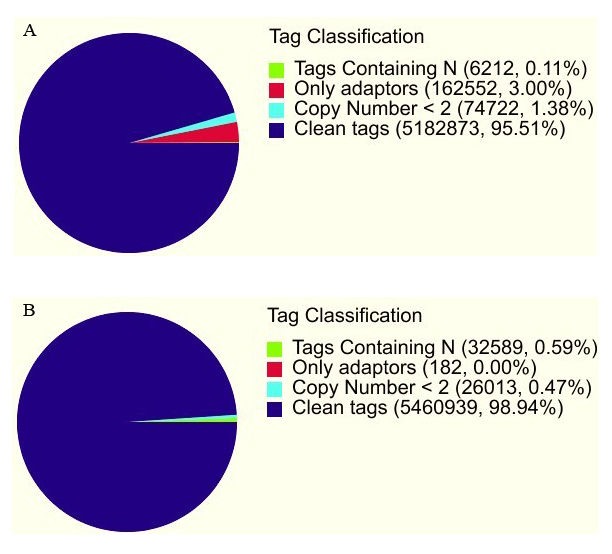

Supplement: Figure S2 — Different components of the raw tags in the two samples. The percentages of the tags containing N, adaptors, a tag copy number<2, clean tags and raw tags. The numbers in parentheses indicate the percentage of each type of tag accounts for the total raw tags. A: trichodermin-producing condition; B: trichodermin-nonproducing condition. (TIF) [file pone.0094203.s002.tif]

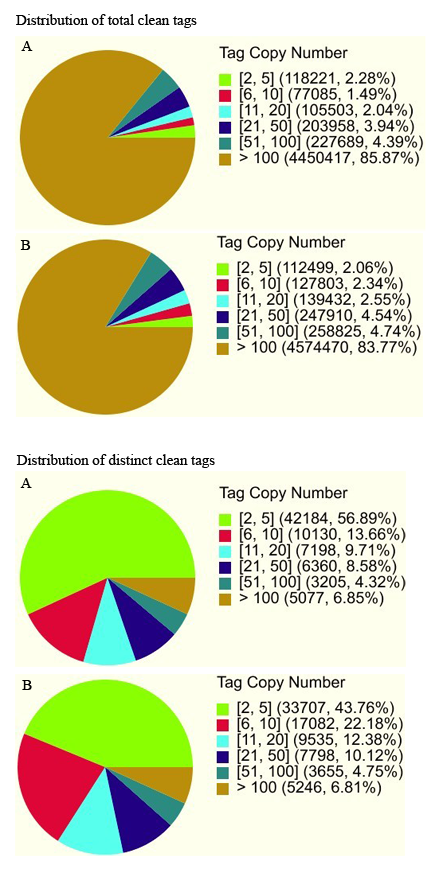

Supplement: Figure S3 — Distribution of the total clean tags and the distinct clean tags in the two samples. The numbers in square brackets indicate the range of copy numbers of each tag category. The data in parentheses indicate the percentage of corresponding tags account for the total clean tags and distinct clean tags. A: trichodermin-producing condition; B: trichodermin-nonproducing condition. (TIF) (TIF) [file pone.0094203.s003.tif]

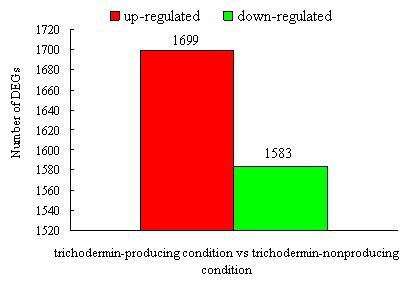

Supplement: Figure S4 — DGE unigenes were up-regulated (red) and down-regulated (green) under the trichodermin-nonproducing or trichodermin-producing condition. (TIF) [file pone.0094203.s004.tif]

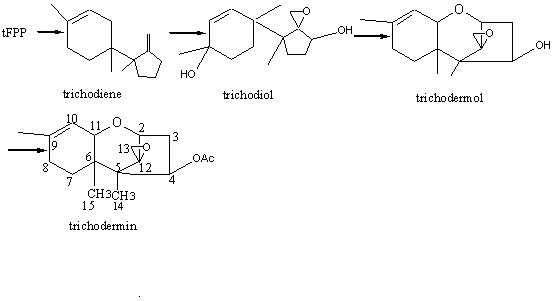

Supplement: Figure S5 — The putative pathway of the trichodermin biosynthesis in Trichoderma brevicompactum . (TIF) [file pone.0094203.s005.tif]

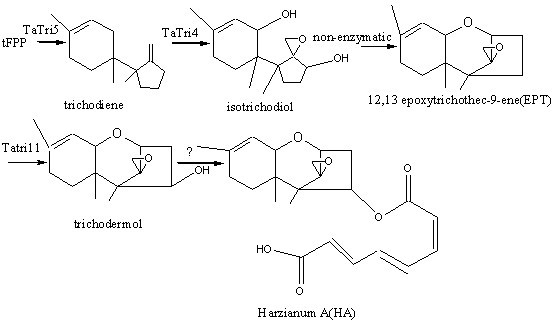

Supplement: Figure S6 — The putative pathway of the HA biosynthesis in Trichoderma arundinaceum . (TIF) [file pone.0094203.s006.tif]
