# Supplementary material for: Transcriptome Sequencing and Gene Expression Analysis of Trichoderma brevicompactum under Different Culture Conditions
Source: PLoS One. 2014 Apr 7;9(4):e94203. doi: 10.1371/journal.pone.0094203 (PMC3978026; doi:10.1371/journal.pone.0094203)
Supplement: Table S4 — Tag analysis statistics. (DOC) [file pone.0094203.s010.doc]

Tag analysis statistics.

| Summary | trichodermin-producing  condition | trichodermin-nonproducing condition |
| --- | --- | --- |
| Raw data | 5,426,359 | 5,519,723 |
| Distinct raw data | 166, 437 | 112,752 |
| Clean tag | 5,182,873 | 5,460,939 |
| Distinct clean tag | 74,154 | 77,023 |
| Clean tag/raw tag | 95.51% | 98.94% |
| All Tag Mapping to Gene | 4,640,258 | 3,602,589 |
| All Tag Mapping to Gene* | 89.53% | 65.97% |
| Distinct All Tag Mapping to Gene | 46,621 | 35,055 |
| Distinct All Tag Mapping to Gene* | 62.87% | 45.51% |
| Unambiguous Tag Mapping to Gene | 3,517,617 | 2,976,946 |
| Unambiguous Tag Mapping to Gene* | 67.87% | 54.51% |
| Distinct Unambiguous Tag Mapping to Gene | 35,647 | 28,361 |
| Distinct Unambiguous Tag Mapping to Gene* | 48.07% | 36.82% |
| Unknown Tag | 542,615 | 1,858,350 |
| Unknown Tag* | 10.47% | 34.03% |
| Distinct unknown tag | 27,533 | 41,968 |
| Distinct unknown tag* | 37.13% | 54.49% |
| Notes:  *% of Clean Tag;  Statistics of raw tags, clean tags, unambiguous tags and unknown tags. | | |
